# Preferences for Analgesic Treatments Are Influenced by Probability of the Occurrence of Adverse Effects and the Time to Reach Maximal Therapeutic Effects

**DOI:** 10.1371/journal.pone.0130214

**Published:** 2015-06-12

**Authors:** Chia-Shu Lin, Shih-Yun Wu, Long-Ting Wu

**Affiliations:** 1 Department of Dentistry, School of Dentistry, National Yang-Ming University, Taipei, Taiwan; 2 Division of Family Dentistry, Department of Stomatology, Taipei Veterans General Hospital, Taipei, Taiwan; 3 Division of Endodontics and Periodontology, Department of Stomatology, Taipei Veterans General Hospital, Taipei, Taiwan; University of Toledo, UNITED STATES

## Abstract

Research on shared medical decision-making suggested that both the potency of a treatment and the probability of it being successful influence individual treatment preferences. Patients also need to consider the negative attributes of treatments, such as the occurrence of adverse effects or a slow start to the therapeutic effects. It remains unclear how these attributes influence individual treatment preferences. We investigated how the analgesic effect, the adverse effect, and the time-course effect influenced the preference of analgesic treatments. Forty-five healthy volunteers participated in three hypothetical analgesic decision-making tasks. They were instructed to imagine that they were experiencing pain and choose between two hypothetical analgesic treatments: the more potent *radical* treatment and the less potent *conservative* treatment. The potency of a treatment was countered by the following attributes: the probability of working successfully, the probability of inducing an adverse effect, and the time required for the treatment to reach its maximal effect. We found that (a) when the overall probability that a treatment would induce an adverse effect decreased, the participants changed their preference from a conservative treatment to a radical treatment; (b) when the time-course for a treatment to reach its maximal effect was shortened, the participants changed their preference from a conservative treatment to a radical treatment, and (c) individual differences in prior clinical pain and the degree of imagined pain relief were associated with preferences. The findings showed that the adverse effects and the time course of treatments guide the analgesic treatment preferences, highlighting the importance of sharing information about negative attributes of treatments in pain management. The findings imply that patients may over-emphasize the occurrence of adverse effect or a slow time-course of treatment effect. In terms of shared medical decision-making, clinicians should clarify these negative attributes related to treatment to patients.

## Introduction

The concept of ‘shared medical decision-making’ has been highlighted in recent years. An essential step in shared decision-making is for clinicians to share with their patients information about treatment benefits and costs and to respond to patients’ treatment preferences [1,2]. Treatment preferences can be guided by information regarding the magnitude of the therapeutic effect and the probability that the effect will occur [3–5]. Regarding pain management, our previous study has investigated the people’s choice between a ‘conservative’ treatment and a ‘radical’ treatment. In the study, the conservative treatment had a weaker potency but a higher probability to work successfully. In contrast, the radical treatment had a stronger potency but a lower probability to work successfully [5]. Our findings revealed that the pain relief that one expected from an analgesic (i.e., the magnitude factor) and the chance that the analgesic worked successfully (i.e., the probability factor) predicted the individual preferences of analgesic treatments [5]. Moreover, the preferences were guided by the magnitude of subjectively expected/imagined pain relief from the analgesic, rather than by the stated amount of potency [5]. These findings are consistent with the predictions based on prospect theory, a descriptive theory of human decision-making [6], and demonstrated that, in terms of treatment benefit, both magnitude and probability play a key role in guiding one’s preferences.

In a real scenario about making decisions to relieve pain, patients may not only consider the treatment benefit (e.g., analgesic effect) but may also consider some ‘negative’ attributes related to the treatment. First, a treatment may induce an unintended and harmful adverse effect [7]. Patients may disfavor a treatment with a higher chance of inducing an adverse effect, even if it is more potent. Second, analgesic treatments usually vary in the time course of the therapeutic effect owing to their pharmacokinetic properties [8]. For example, some treatments start to work more slowly than others, but their effects last for a longer period of time. Patients may disfavor such a slowly starting treatment, even if it is more potent in the long term. How the adverse effects and time course of a treatment influence the analgesic treatment preference have remained unknown. According to prospect theory, a decision-maker would asymmetrically overweight the aggravation from losses, compared to the pleasure from gains [6]. Therefore, people would prefer avoiding losses to obtaining gains [6,9]. In the clinical scenario, patients usually need to balance the benefits from a treatment and the potential risk of an adverse effect. We reasoned that the sense of loss aversion, i.e. to avoid the occurrence of an adverse effect, would be a critical factor that guides analgesic decision-making. The effect of time-course can be framed as making a intertemporal choice, where a decision-maker needs to tradeoff between the benefits and the temporal delay to get the benefits [9]. The discounted utility model predicts that decision-makers would discount the value of an outcome delayed in time, and become short-sighted in making decisions [10]. Critically, such a short-sighted behavior may be more prominent with the decision is related to visceral needs, such as thirsty, hunger and pain [11]. Therefore, we reasoned that the interval for an analgesic treatment to reach its maximal effect would guide one’s preference, during analgesic decision-making.

Additionally, the degree of pain relief varied greatly across individuals [5]. A possible reason for such a variation is the individual difference in psychological traits about pain. The pain-related psychological factors may include:

1. *Prior clinical pain*: The perception of pain can be influenced by one’s prior experience of pain [12,13]. For example, a participant who had suffered from an extremely painful headache may be more enthusiastic about getting an analgesic relief, compared to a participant who has no such experience.
2. *The degree of imagined pain relief*: Our previous findings showed that imagined pain relief to the stated analgesic potency varied across participants [5]. The degree of expected or imagined relieving potency from an analgesic treatment, as the monetary gain in financial decision-making, would guide one’s preference [6].
3. *Reward-seeking*: The experience of getting pain relieved can be considered as a kind of psychological reward [14,15]. In health-related decision-making, the propensity towards fun-seeking, as assessed by the Behavioral Activation Scale (BAS), was positively associated with a preference for risk-seeking choices [16,17]. We reasoned that the difference the attitude about seeking reward or pleasure would influence the analgesic decision-making.
4. *Fear of pain / Pain catastrophizing*: Previous studies have revealed that the degree of the fear of pain was positively correlated with decisions to avoid painful stimuli [18]. The trait of pain catastrophizing also plays a key role in fear-avoidance behavior around acute and chronic pain [19,20]. Therefore, an individual with a higher degree in these traits may be enthusiastic about getting a relief, so as to reduce the pain-related fear.

In the present study, we assessed preferences related to pain management using an analgesic decision-making task [5] that was based on previous research on health-related and financial decision-making [3–6]. While the previous study focused on the benefit domain (i.e., the probability that a treatment works successfully) of analgesic treatment [5], the current study focused on the negative domain (i.e., an adverse effect and time-course of a treatment) of medical decision-making. In different hypothetical scenarios (Table 1), the participants were asked to imagine that they were in pain and to choose between two hypothetical analgesic treatments that differed in the analgesic effect and other attributes. We systematically tested the following three hypotheses:

1. The overall probability that a treatment will induce an adverse effect influences analgesic treatment preferences. Specifically, when the overall probability that a treatment would induce an adverse effect decreases, the participants will change their preferences from a conservative treatment to a radical treatment.
2. The time interval for a treatment to reach its maximal effect (i.e. the time-course) influences analgesic treatment preferences. Specifically, when the time-course is shortened, the participants will change their preferences from a conservative treatment to a radical treatment.
3. The association between the psychological traits and the analgesic treatment preferences has not been elucidated. We tested the hypothesis that pain and psychological factors, including (a) the ratings of the mean prior clinical pain, (b) the degree of imagined pain relief, and the degree of (c) reward-seeking, (d) fear of pain, and (e) pain catastrophizing, would account for the individual variance in preference.

**Table 1:** Design of the hypothetical decision-making scenarios.

**(A) ‘Analgesic Effect’ task: Potency of the treatment**				
ID	ΔP	Overall probability	Conservative	Radical
1	Smaller	90%	Potency: ΔP9→6 Probability: 90%	Potency: ΔP9→3 Probability: 45%
2	Smaller	30%	Potency: ΔP9→6 Probability: 30%	Potency: ΔP9→3 Probability: 15%
3	Smaller	0.2%	Potency: ΔP9→6 Probability: 0.2%	Potency: ΔP9→3 Probability: 0.1%
4	Larger	90%	Potency: ΔP9→6 Probability: 90%	Potency: ΔP9→0 Probability: 45%
5	Larger	30%	Potency: ΔP9→6 Probability: 30%	Potency: ΔP9→0 Probability: 15%
6	Larger	0.2%	Potency: ΔP9→6 Probability: 0.2%	Potency: ΔP9→0 Probability: 0.1%
**(B) ‘Adverse Effect’ task: Potency of the treatment**				
ID	ΔP	Overall probability	Conservative	Radical
7	Smaller	90%	Potency: ΔP9→6 Probability: 45%	Potency: ΔP9→3 Probability: 90%
8	Smaller	30%	Potency: ΔP9→6 Probability: 15%	Potency: ΔP9→3 Probability: 30%
9	Smaller	0.2%	Potency: ΔP9→6 Probability: 0.1%	Potency: ΔP9→3 Probability: 0.2%
10	Larger	90%	Potency: ΔP9→6 Probability: 45%	Potency: ΔP9→0 Probability: 90%
11	Larger	30%	Potency: ΔP9→6 Probability: 15%	Potency: ΔP9→0 Probability: 30%
12	Larger	0.2%	Potency: ΔP9→6 Probability: 0.1%	Potency: ΔP9→0 Probability: 0.2%
**(C) ‘Time-course Effect’ task**				
ID	ΔP	Number of days	Conservative	Radical
13	Smaller	5 days	Potency: ΔP9→3 constantly for 7 days	Potency: ΔP9→6 for the first 5 days and then ΔP9→0
14	Smaller	3 days	Potency: ΔP9→3 constantly for 7 days	Potency: ΔP9→6 for the first 3 days and then ΔP9→0
15	Smaller	1 day	Potency: ΔP9→3 constantly for 7 days	Potency: ΔP9→6 for the first 1 days and then ΔP9→0
16	Larger	5 days	Potency: ΔP9→6 constantly for 7 days	Potency: no relief for the first 5 days and then ΔP9→0
17	Larger	3 days	Potency: ΔP9→6 constantly for 7 days	Potency: no relief for the first 5 days and then ΔP9→0
18	Larger	1 day	Potency: ΔP9→6 constantly for 7 days	Potency: no relief for the first 5 days and then ΔP9→0

## Methods and Materials

### Participants

Forty-five healthy, pain-free participants [25 women, age 30.7±10.4, (the mean ± standard deviation)] participated in this study. None of the participants had a history of chronic pain or had been previously diagnosed with a psychiatric disorder (see Table 2 for the demographic profiles of the participants). All participants were recruited via the advertisement posted around the university campus. The participants would obtain reimbursement for transportation when they finished the experiment. All of the participants completed an undergraduate degree (Table 2). The homogeneity of education level suggested that all participants were capable of performing the hypothetical decision-making tasks, which demands basic degree of numeracy. All of the participants provided written informed consent before participating in this study. The study protocol was approved by the Institutional Review Board of Taipei Veterans General Hospital. The participants were recruited in two waves: 37 participants were recruited from the workers or researchers in a university and 8 participants were recruited from the volunteer workers in a hospital. The sample size was determined according to statistical power analysis (for details see S1 Text).

**Table 2:** Demographic data and prior experience of clinical pain.

	Total (N = 45)	Male (n = 20)	Female (n = 25)
Age (Years)	30.7 (10.4) ^1^	31.9 (11.5)	29.8 (9.5)
Range	22–54	22–54	22–52
Education level (Years)	17.4 (1.0)	17.2 (1.1)	17.6 (0.9)
Range	16–19	16–19	16–19
N Recent clinical pain ^2^	1	1	0
N Use of analgesics ^3^	1	0	1
Maximal intensity of past clinical pain (0–10) ^4^			
Toothache	6.8 (1.8)	6.9 (1.7)	6.7 (1.6)
Headache	7.3 (2.1)	6.7 (2.1)	7.7 (2.1)
Stomach ache	6.4 (2.6)	5.6 (3.1)	7.1 (2.1)
Pain and psychological factors			
FPQ-III	28.3 (5.2)		
PCS	24.8 (13.1)		
BAS	49.8 (8.0)		

^1^Values between brackets are standard deviations.

^2^One male participant had taken analgesic within one month before the study, due to a sore throat.

^3^One female participant had taken analgesics due to migraine for more than one year. The migraine had ceased to occur at the time of the study.

^4^Maximal intensity of clinical pain were made on 0–10 numerical rating scale (0 = no pain, 10 = extremely painful).

### Prior Experience of Clinical Pain

In the analgesic decision-making task, we assessed the participants’ analgesic treatment preferences in hypothetical scenarios where they imagined that they were in pain [5]. Because the intensity of imagined pain and pain relief can be influenced by one’s prior experience of pain [12,13], we performed the following two investigations for assessing their prior experience of clinical acute pain. First, the participants reported if they had visited a medical provider due to clinical pain (such as a toothache or headache) within the last month and if they had continuously used analgesics for more than one year before they participated in the study. Second, they rated their most intense prior experience of clinical pain, respectively for toothaches, headaches and stomach aches, using a 11-point numerical rating scale (0 = no pain, 10 = worst possible pain). We focused on these experiences because they are common occurrences in healthy participants. In addition, because the intensities of toothaches, headaches and stomach aches can be moderate to intense, the participants would have a stronger motivation to relieve this pain.

### Rating Task of Imagined Pain Relief

We explicitly distinguished between the *stated potency* of an analgesic treatment, and the *pain relief* that one would experience from the treatment. We defined *stated potency*, i.e. the analgesic effect, as the amount of pain reduction explicitly labeled or told by clinicians. Stated potency is usually described in a relative sense, such as a decrease in pain intensity from 9 (or ‘strong pain’) to 3 (or ‘mild pain’), based on a 0–10 numerical rating scale. Therefore the degree of stated potency is denoted by ΔP. In contrast, *pain relief* is a psychological construct that reflects the hedonic experience or pleasure related to pain reduction [14]. It should be noted that, across individuals, the degree of pain relief may vary greatly to a fixed level of stated potency [5]. For example, while some patients would regard the analgesic potency ΔP9→6 (i.e., reduction of pain from 9 to 6) as very pleasant and satisfied, others may regard it ‘not good enough’ and do not feel their pain relieved. We considered such an individual difference a critical variable that would guide the preferences for analgesic treatments.

In a real clinical scenario, when making a treatment-related decision, patients usually imagine the therapeutic effect of a treatment before they actually experience its effect. To assess the imagined pain relief [9] of stated potency, we performed a hypothetical rating task before the decision-making tasks. We asked the participants to imagine that they were experiencing an acute pain (toothache, headache and stomach ache), with an intensity of 9. The intensity was based on a 0–10 numerical rating scale (NRS) (0 = non-painful and 10 = extremely painful). Stated potency was defined as the amount of pain reduction (ΔP). For example, Δ9→0 denotes that the treatment would relieve pain from 9 to 0. We next asked the participants to rate the degree of imagined relief about stated potency (indexed as *imagined pain relief*, [5]) for the following hypothetical treatments: (1) a treatment that reduced pain from 9 to 0 (denoted by Δ9→0), (2) a treatment that reduced pain from 9 to 6 (Δ9→6), and (3) a treatment that reduced pain from 9 to 3 (Δ9→3). The stated potency Δ9→6 and Δ9→3 were chosen because they were associated with a greater individual differences in imagined pain relief, based on our previous study [5]. The rating of imagined pain relief was based on a 0–100 scale (0 = no relief and 100 = the strongest relief). They were asked to perform the rating task for a toothache, headache and stomach ache.

### Design of Analgesic Decision-making Task

The participants completed a pencil-and-paper questionnaire that consisted of three analgesic decision-making tasks (the ‘*Analgesic Effect*’ task, the ‘*Adverse Effect*’ task, and the ‘*Time-course Effect*’ task). Each task consisted of six scenarios, and therefore all participants needed to complete 18 scenarios in total (see Table 1 for the detailed description about each scenario). In each scenario, the participants were instructed to imagine that they were experiencing pain at level 9, based on the same NRS in the rating task, and they then needed to choose between two analgesic treatments to reduce the pain. Within a scenario, the two treatments differed in their stated potency.

Between these two hypothetical treatments, we designated the treatment that was more potent as the *radical* treatment and the treatment that was less potent as the *conservative* treatment. Importantly, the potency of a treatment was countered by a treatment-related attribute.

1. In the ‘Analgesic Effect’ task, the participants needed to tradeoff between the treatment potency and its probability of working successfully. The radical treatment had a lower overall probability of working successfully. As an example (Scenario 1 of Table 1), the participants were asked to choose between two hypothetical analgesic treatments for relieving their pain. The conservative treatment reduces pain from 9 to 6 (i.e., Δ9→6) if it works (probability 90%); the radical treatment reduces pain from 9 to 3 (i.e., Δ9→3) if it works (probability 45%).
2. In the ‘Adverse Effect’ task, the participants needed to tradeoff between the treatment potency and the occurrence of an adverse effect. The radical treatment had a higher probability of inducing an adverse effect. As an example, (Scenario 7 of Table 1), the participants were asked to choose between two hypothetical analgesic treatments for relieving their pain. The conservative treatment reduces pain from 9 to 6 (i.e., Δ9→6) and an adverse effect may occur (probability 45%); the radical treatment reduces pain from 9 to 3 (i.e., Δ9→3) and an adverse effect may occur (probability 90%).
3. In the ‘Time-course Effect’ task, the radical treatment was slower to reach its maximal analgesic effect. In this task, the participants needed to tradeoff between the treatment potency and the time-course for a treatment to reach its maximal effect. As an example (Scenario 13 of Table 1), the participants were asked to choose between two hypothetical analgesic treatments for relieving their pain. The conservative treatment reduces pain from 9 to 3 (i.e., Δ9→3) constantly for 7 days; the radical treatment reduces pain from 9 to 6 (i.e., Δ9→6) for the first 5 days and then it reduces pain from 9 to 0 (i.e., the maximal effect) from the 6th day.

Across the participants, the order of the ‘Adverse Effect’ task and the ‘Time-course Effect’ task were counterbalanced. Twenty-two participants took the experiment in the order ‘Analgesic—Adverse Effect—Time-course’ (i.e. AE-TC), and 23 participants took the experiment in the order ‘Analgesic—Time-course—Adverse Effect’ (i.e. TC-AE). For each task, the preference did not significantly differ between the two groups (see S1 Text for detailed results).

#### The ‘Analgesic Effect’ task

In this task, the scenarios differed in the overall probability that a treatment would work successfully (i.e., have the expected analgesic effect). We manipulated the overall probability at three discrete levels: 90%, 30% and 0.2% (Table 1A). These probabilities indicated the chance that the conservative treatment would work successfully, and the chance of the radical treatment working successfully was always half of the overall probability. For example, when the overall probability was set to 90%, the probabilities of the conservative and the radical treatments working successfully were 90% and 45%, respectively. In brief, the probability of a treatment working successfully and its potency acted in opposition in the treatments (Table 1A): the *radical* treatment was always more potent but less likely to work successfully, and the *conservative* treatment always was less potent but more likely to work successfully.

#### The ‘Adverse Effect’ task

In this task, the scenarios differed in the overall probability that a treatment would induce an adverse effect. We manipulated the overall probability at three discrete levels: 90%, 30% and 0.2% (Table 1B). These probabilities indicated the chances that the radical treatment would induce an adverse effect, and the chance of the conservative treatment inducing an adverse effect was always half of the overall probability. In brief, the probability for a treatment to induce an adverse effect and its potency acted in opposition in the treatments (Table 1B): the *radical* treatment was always more potent but more likely to induce an adverse effect, and the *conservative* treatment was less potent but less likely to induce an adverse effect.

#### The ‘Time-course Effect’ task

In this task, the scenarios differed in the time course of the analgesic effect. We quantified the time-course as the interval (number of days) between the time when a patient received the treatment and the time when the treatment reached its maximal effect. We manipulated this time interval at three levels: 1 day, 3 days and 5 days (Table 1C). We only manipulated the time-course of the radical treatment, while the time-course of the conservative treatment was held constant. For example, when the interval was ‘3 days’, the radical treatment reduced pain from 9 to 6 over the first 3 days, and then further reduced it to 0 over the next 4 days (i.e., it reached its maximal effect after 3 days). In contrast, the conservative treatment reduced pain from 9 to 3 immediately and kept the same effect for 7 days. In brief, the time interval for a treatment to reach its maximal effect and its potency acted in opposition in the treatments (Table 1C): the *radical* treatment was always more potent over the long run but was slower to reduce pain, and the *conservative* treatment was less potent over the long run but was quicker to reduce pain.

#### The effect of relative potency between treatments

Our previous study showed that the relative potency between two treatments, as a magnitude factor, influences individual preferences [5]. We here manipulated the magnitude factor at two levels. In each task, the scenarios differed in relative potency (i.e., ΔP of the radical treatment / ΔP of the conservative treatment). For each task, three scenarios were assigned as having a ‘smaller relative ΔP’ (i.e., Scenario 1–3, 7–9 and 13–15 of Table 1). In these scenarios, the potencies of the conservative treatments and the radical treatments were ΔP9→6 and ΔP9→3, respectively. In the other three scenarios (i.e., Scenario 4–6, 10–12 and 16–18 of Table 1), the potencies of the conservative treatments and the radical treatments were ΔP9→6 and ΔP9→0, respectively. Note that the potency of the conservative treatment was used as a reference for comparison. We referred to its potency (ΔP9→6) as *referential pain relief*, which the analgesic effect of the radical treatment was compared with.

### Psychological Assessment

The participants were asked to complete the Fear of Pain Questionnaire-III (FPQ-III, [21]) and the Pain Catastrophizing Scale (PCS, [22]), which are associated with pain-related fear and maladaptive cognition about pain. Additionally, the Behavioral Activation Scale (BAS) from the Behavioral Inhibition/Behavioral Activation Scales (BIS/BAS, [23]) was used to assess the reward-seeking traits of the participants. All of the questionnaires were translated to Chinese, according to their corresponding Chinese versions (FPQ-III: [24], PCS: [25], BAS: [26]).

### Statistical Analysis

All of the statistical procedures were performed using the statistical software package MedCalc (MedCalc Software, Ostend, Belgium) and PASW Statistics for Windows (Version 18.0, Chicago: SPSS Inc).

#### Prior experience of clinical pain and imagined pain relief

**Fig 1:**
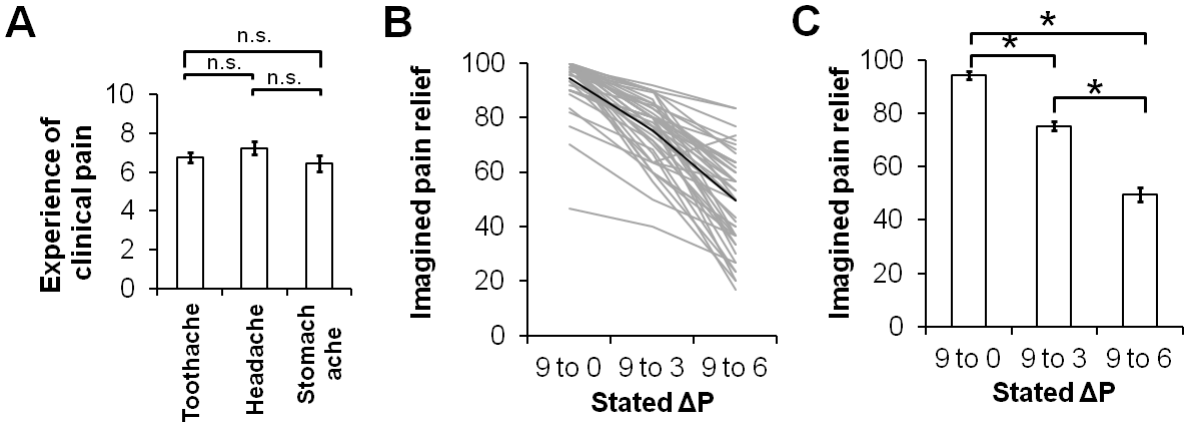
Analysis of prior experience of clinical pain and the ratings of imagined pain relief. **(A)** The one-way ANOVA of repeated measures shows no significant difference in the maximal intensity of prior pain between the three sources of clinical pain. **(B)** All the participants show a pattern of decreased imagined pain relief, as the treatment potency (as shown in the x-axis) decreases. The thick line represents the averaged imagined pain relief from all participants. **(C)** The one-way ANOVA of repeated measures shows significant differences between the three levels of treatment potency. n.s., non-significant; asterisk denotes statistical significant P <0.001.

For prior experiences of clinical pain, we performed a one-way ANOVA of repeated measures to compare the ratings of prior pain experiences between toothaches, headaches and stomach aches. Because the result did not show a significant difference in prior experiences between the pain sources (see Results and Fig 1A), in the subsequent analysis, we averaged the ratings from the three types of pain, and the resulting index *mean of prior clinical pain* was used in the regression analysis. The ratings of imagined pain relief were also averaged across the three types of clinical pain separately for the conditions Δ9→6, Δ9→3 and Δ9→0. We then performed a one-way ANOVA of repeated measures to compare the averaged ratings of imagined pain relief between the conditions Δ9→6, Δ9→3 and Δ9→0. A post-hoc Student-Newman-Keuls test was performed for the pairwise comparison of subgroups.

#### Hypothesis 1 and 2: Analysis of group preferences

In each scenario of the tasks, we quantified the participants’ preferences at the group level as the percentage of the participants who chose the radical treatment, denoted as *Choice_radical_%*. A higher value of Choice_radical_% indicated that more participants chose the radical treatment in that scenario. To compare Choice_radical_% between the six scenarios in each task, we applied Cochran's Q test, a non-parametric statistical approach that tests for differences in frequencies between three of more related pieces of data. The test is commonly used for comparing multiple-paired binary outcomes, such as the results of a diagnosis (e.g., [27]), and in the present study, the preferences of treatments (i.e., radical or conservative). We also performed a comparison between each pair of the six scenarios for each task. It should be noted that multiple comparisons ([6 X 5 / 2] = 15 comparisons in total) were made simultaneously; therefore, the significance level was adjusted as 0.05/15 = 0.003, according to Bonferroni correction for multiple comparison.

Based on our first and second hypotheses, we expected that Choiceradical% varied between different conditions of overall probability (for the ‘Adverse Effect’ task) and between different the time interval for a treatment to reach its maximal effect (for the ‘Time-course Effect’ task).

### Hypothesis 3: Analysis on the effect of pain and psychological factors on preference

To understand if pain and psychological factors predict the participants’ preferences, we performed a logistic regression analysis, with a force-entry model, by taking the preference of treatment (‘conservative’ vs. ‘radical’) as the dependent variable, and the following four factors as the independent variables (i.e. predictors): (a) the ratings of the mean prior clinical pain, (b) the ratings of referential pain relief (Δ9→6), and the scores of the (c) BAS and (d) PCS.

It should be noted that the logistic regression analysis was performed only for the following scenarios: Scenario 1, 2, 10 and 16 (see Results). The four scenarios were selected because the frequency of choosing a conservative or a radical treatment was more balanced (between 40% and 60%), which would be more consistent with the clinical condition that patients divert in their decisions. Because we tested four conditions simultaneously, the significance level of each logistic regression model was adjusted as 0.05/4 = 0.012, according to Bonferroni correction for multiple comparison.

#### Analysis of the individual patterns of preferences

We further investigated the individual patterns of preferences at different levels of overall probability and time intervals for treatments to reach their maximal effects. Because we graded each attribute into three levels, we marked the pattern of preferences with a triplet of letters consisting of ‘C’ (for choosing a conservative treatment) or ‘R’ (for choosing a radical treatment). In the ‘Analgesic Effect’ and the ‘Adverse Effect’ tasks, the three letters indicated a participant’s preference when the overall probability was 90%, 30% and 0.2%. For example, ‘CRR’ indicated that the participant chose a conservative treatment when the probability was 90% and then shifted to the radical treatment when the probability was 30% and 0.2%. Likewise, in the ‘Time-course Effect’ task, the three letters indicated the preference when the duration was 5 days, 3 days and 1 day. Therefore, there were six possible patterns in each scenario (‘CCC’, ‘CCR’, ‘CRR’, ‘RCC’, ‘RCR’, and ‘RRR’). Here we only analyzed the following four patterns ‘CCC’, ‘CCR’, ‘CRR’, and ‘RRR’, which represented a transition of the preference pattern from more conservatism to more radicalism. Across all the participants, we calculated the percentage of the participants who adopted each of the four patterns. The analysis was performed for both the ‘smaller relative ΔP’ and the ‘larger relative ΔP’ scenarios, respectively. A pattern was defined as the ‘dominant pattern’ in the scenario, if more than 50% of the participants adopted that pattern.

Additionally, to assess whether the pain or psychological factors would influence the choice pattern, we performed an ordered multimodal regression analysis, with a force-entry model, by taking the four patterns as the dependent variables and the following four factors as the independent variables (i.e. predictors): (a) the ratings of the mean prior clinical pain, (b) the ratings of referential pain relief (Δ9→6), and the scores of (c) the BAS and (d) the PCS. The four patterns were conceptualized as an ordinal scale which representing the transition from conservatism to radicalism (‘CCC’ = total conservatism and ‘RRR’ = total radicalism).

## Results

### Prior Experience of Clinical Pain and Imagined Pain Relief

For experiences of clinical pain, a one-way ANOVA of repeated measures showed no significant difference in the ratings of prior clinical pain between headache, toothache and stomach ache (F2,88 = 1.62, P = 0.08) (Fig 1A). The ratings of headache were significantly correlated with the ratings of stomach ache (Pearson’s correlation coefficient r = 0.39, P = 0.009). For imagined pain relief, a one-way ANOVA of repeated measures showed a significant difference in the ratings of averaged imagined pain relief between the conditions (F2,88 = 109.2, P <0.001) (Fig 1B and 1C). The subsequent post-hoc analyses showed that the ratings in Δ9→0 (94.3±10.0) were significantly higher than those in Δ9→3 (75.3±11.6) (P <0.05), and the ratings in Δ9→3 were significantly higher than those in Δ9→6 (49.4±17.8) (P <0.05) (Fig 1C). This relationship of imagined pain relief (i.e., Δ9→0 > Δ9→3 > Δ9→6) was highly consistent among all participants (Fig 1B).

### Analysis of group preferences

#### The ‘Analgesic Effect’ task

**Fig 2:**
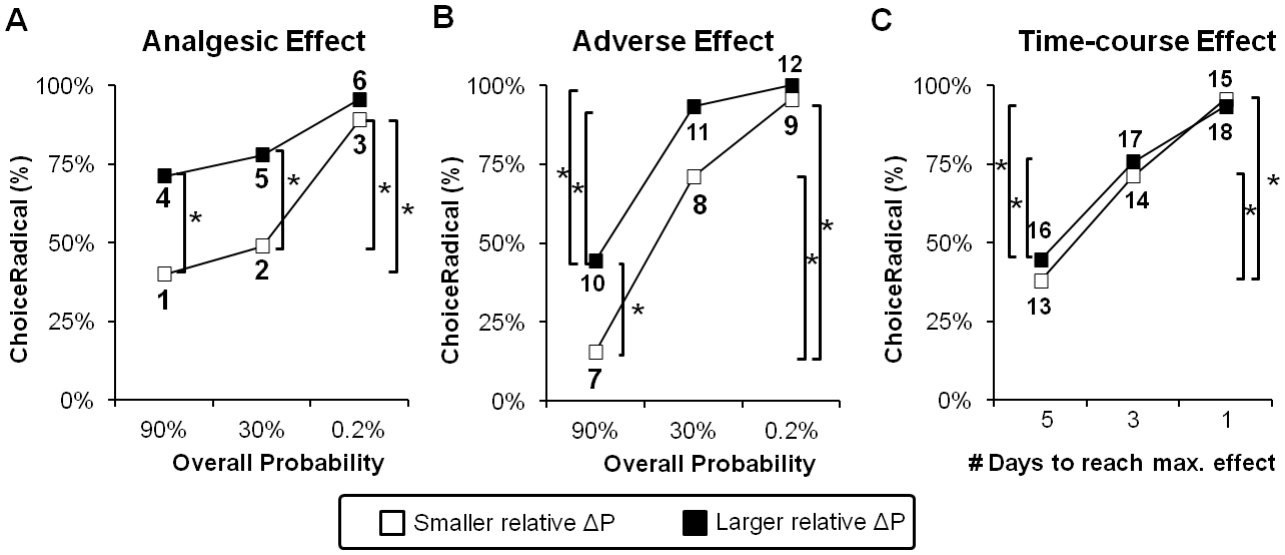
Analysis of preference at the group level . **(A)** Results from the ‘Analgesic Effect’ task. **(B)** Results from the ‘Adverse Effect’ task. **(C)** Results from the ‘Time-course Effect’ task. The y-axis denotes the percentage of participants who chose the radical treatment. The x-axis denotes the three levels of overall probabilities of the treatments working successfully, the overall probability of inducing an adverse effect, and the number of days for the treatment to reach maximal effect, respectively. The number aside the square indicates the scenario ID, as shown in Table 1.

The Cochran’s Q test showed that the frequency for the participants to choose a radical treatment was significantly different between the six scenarios (Cochran’s Q = 55.6, P<0.0001). Between each pair of scenarios, we found a significant difference in Choiceradical% between the levels of the overall probability (i.e., 90% vs. 0.2% and 30% vs. 0.2%) in the scenarios of smaller relative ΔP. As the overall probability increased, the proportion of participants who chose the radical treatment increased (Fig 2A, open squares). In contrast, in the scenarios with a larger relative ΔP, Choiceradical% did not significantly differ between the levels of overall probability (Fig 2A, solid squares). We also found a significant difference in Choiceradical% between the scenarios with the smaller and larger relative ΔPs when the overall probability was 90% and 30%. The results were consistent with the findings from our previous study, which showed that both the magnitude and the probability factors influenced the preference of analgesics [5].

#### The ‘Adverse Effect’ task

The Cochran’s Q test showed that the frequency for the participants to choose a radical treatment was significantly different between the six scenarios (Cochran’s Q = 118.5, P<0.0001). Between each pair of scenarios, we found a significant difference in Choiceradical% between the levels of overall probability (i.e., 90% vs. 30% and 90% vs. 0.2%) in both the scenarios of smaller relative ΔP (Fig 2B, open squares) and larger relative ΔP (Fig 2B, solid squares). We also found a significant difference in Choiceradical% between the scenarios with the smaller and larger relative ΔPs when the overall probability was 90%. The findings confirmed our first hypothesis, demonstrating that the overall probability that a treatment will induce an adverse effect would influence analgesic treatment preferences.

#### The ‘Time-course Effect’ task

The Cochran’s Q test showed that the frequency for the participants to choose a radical treatment was significantly different between the six scenarios (Cochran’s Q = 70.4, P<0.0001). Between each pair of scenarios, we found a significant difference in Choiceradical% between the levels of the time-courses (i.e., 5 days vs. 3 days and 5 days vs. 1day) in both the scenarios of smaller relative ΔP (Fig 2C, open squares) and larger relative ΔP (Fig 2C, solid squares). We did not find a significant difference in Choiceradical% between the scenarios of smaller and larger relative ΔPs. The findings confirmed our second hypothesis that the time-course (the time interval for a treatment to reach its maximal effect) influence analgesic treatment preferences.

### Analysis on the Effect of Pain and Psychological Factors on Preferences

Regarding the psychological factors about pain, all three scales showed a high internal consistency (Table 2). The FPQ-III score was significantly correlated with the PCS score (r = 0.42, P <0.01) (Table 2). Because the strong collinearity between the FPQ and the PCS scores, we only considered the PCS score as the independent variable in the subsequent regression analysis.

For the ‘Time-course Effect’ task (Scenario 16), the logistic regression analysis showed that among the four independent variables (the ratings of the mean prior clinical pain, the ratings of referential pain relief (Δ9→6), and the scores of the BAS and the PCS, only the variable ‘mean prior clinical pain’ was significantly associated with individual preference (β = -0.90, Exp(B) = 0.40, P = 0.006). When the ratings of prior clinical pain increased, the odds for the participants to choose a radical treatment, over a conservative treatment, would decline, after controlling other three variables.

In contrast, for the ‘Analgesic Effect’ task (Scenario 1 and 2) and the ‘Adverse Effect’ task (Scenario 10), none of the independent variables showed significant association with preferences.

### Individual Patterns of Preferences

#### The ‘Analgesic Effect’ task

**Fig 3:**
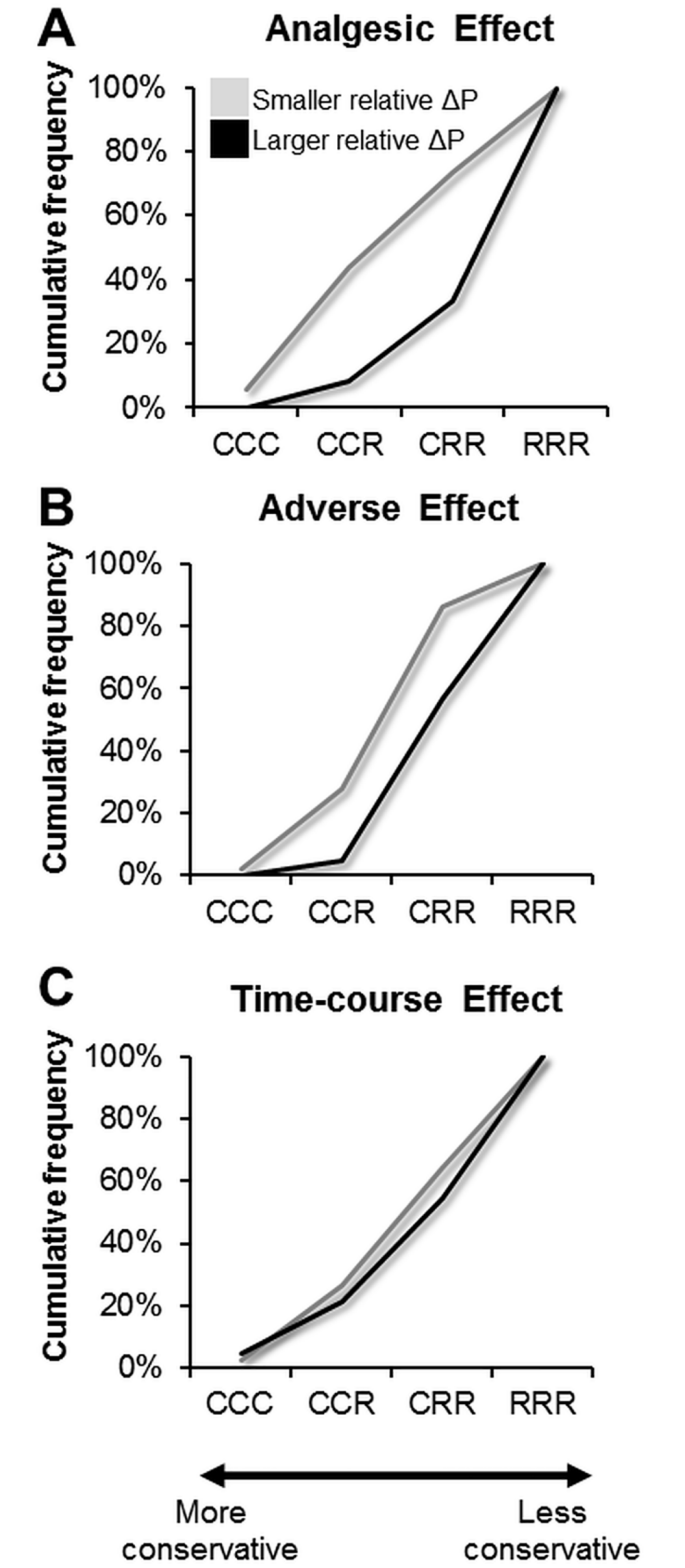
Analysis of the pattern of preference. **(A)** Results from the ‘Analgesic Effect’ task. **(B)** Results from the ‘Adverse Effect’ task. **(C)** Results from the ‘Time-course Effect’ task. On the x-axis, the three letters denote the preference (radical or conservative, i.e., R or C) corresponding to the three levels of probability (90%, 30% and 0.2%) or the number of days (5 days, 3 days and 1 day). The patterns of preference is arranged in the order that represents the degree of conservatism, ranging from ‘less conservative’ (i.e. the pattern ‘CCC’) to ‘more conservative’ (i.e. the pattern ‘RRR’). The y-axis denotes the cumulative proportion of the participants who adopted this pattern of preference.

When the relative ΔP of the two treatments was smaller, we did not find a predominant pattern of preferences among the participants (Fig 3A). The patterns ‘CCR’, ‘CRR’ and ‘RRR’ were respectively adopted by 38%, 29% and 27% of the participants. In contrast, when the relative ΔP was larger, we found the pattern ‘RRR’ dominated, which was adopted by 67% of the participants. This pattern indicated that most of the participants adopted the radical treatment, regardless of the levels of overall probability (Fig 3A).

Additionally, the ordered multimodal regression analysis showed that among the four independent variables (the ratings of the mean prior clinical pain, the ratings of referential pain relief (Δ9→6), and the scores of the BAS and PCS.), only the variable ‘referential pain relief’ was significantly associated with the patterns of preference (β = -0.065, P = 0.032), in the scenarios of the ‘Analgesic Effect’ task with large relative ΔP. The finding revealed that when referential pain relief increased, the odds for the participants to choose a more radical pattern, over a more conservative pattern, would decline, after controlling the other three variables. In contrast, for the ‘Adverse Effect’ task and the ‘Time-course Effect’ task, none of the independent variables showed significant association with the patterns of preference.

#### The ‘Adverse Effect’ task

We found the predominant pattern of preference was ‘CRR’ (Fig 3B), which was adopted by 58% and 52% of the participants in the scenarios of small and large relative ΔP, respectively. Notably, in the scenario of a large relative ΔP, a larger percentage of the participants (43%) adopted the pattern ‘RRR’ (Fig 3B).

#### The ‘Time-course Effect’ task

We did not find a predominant pattern of preferences among the participants (Fig 3C). When the relative ΔP of the two treatments was smaller, the patterns ‘CCR’, ‘CRR’ and ‘RRR’ were respectively adopted by 24%, 38% and 36% of the participants. When the relative ΔP of the two treatments was larger, the patterns ‘CCR’, ‘CRR’ and ‘RRR’ were respectively adopted by 17%, 33% and 45% of the participants.

## Discussion

### Summary of Major Findings

Previous studies of medical decision-making have focused on the therapeutic effects of treatment. These findings suggest that both the potency of a treatment and the probability of it working successfully may influence individual treatment preferences [3–5]. In terms of treatment-related decision-making, patients often need to consider some attributes related to a treatment, such as whether an adverse effect may occur or how quickly the therapeutic effect begins to work. It has remained unclear how these attributes influence individuals’ preferences of treatments. Here we systematically investigated how the analgesic effect, the adverse effect, and the time-course effect influenced the preference of analgesic treatment. Our major findings include:

1. The overall probability that a treatment will induce an adverse effect influences analgesic treatment preferences. Specifically, when the overall probability that a treatment would induce an adverse effect decreased, the participants changed their preference from a conservative treatment to a radical treatment.
2. The time interval for a treatment to reach its maximal effect (i.e. the time-course) influences analgesic treatment preferences. Specifically, when the time-course was shortened, the participants changed their preference from a conservative treatment to a radical treatment.
3. A higher degree of prior clinical pain was associated with a lower probability to choose the radical treatment, in the ‘Time-course’ task. A higher degree of imagined pain relief was associated with a lower probability to form a radical pattern of preferences, in the ‘Analgesic Effect’ task.

Our previous study has shown that information about the benefit domain of therapeutic effect would influence one’s preferences of analgesic treatment. We here extended the findings, showing that information about the negative domain—the occurrence of adverse effect and the time-course of treatment effect—both influence one’s preferences of analgesic treatment.

### The Adverse Effect on Analgesic Decision

Our results showed that the overall probability played a key role in the preference of both the analgesic and the adverse effects (Fig 2A and 2B). At the individual level, most of the participants shifted their preference from a conservative treatment to a radical treatment as the overall probability decreased. This pattern of preference is consistent with the prediction of prospect theory [6]. Prospect theory has offered a descriptive framework of how individuals make choices under risk [6]. (The term ‘risk’ here denotes the degree of unpredictability regarding obtaining an outcome [28]). According to prospect theory, preferences are associated with *decision weight*, namely, the psychological impact of the stated probability of an outcome’s occurrence [6]. The subjective decision weight is nonlinearly associated with the objectively stated probability. For example, when the overall probability is very small (e.g., in our task, 0.2% vs. 0.1%), individuals tend to overweight the small probabilities. Our study therefore demonstrated that the probability factor plays a key role for individuals in integrating either benefit- or risk-related information about a treatment during decision-making.

Interestingly, we found an asymmetric effect of overall probability on the analgesic and adverse effects. For the analgesic effect, not only the overall probability level but also the relative potency between treatments (i.e., the magnitude effect) influenced one’s preference (Fig 2A). The results confirmed our previous finding that the effect of overall probability was more prominent when the relative potency was smaller. In contrast, when the relative potency was larger, the participants consistently chose the radical treatment, regardless of the overall probability levels (Fig 2A). For the adverse effect, the pattern of preferences was less influenced by such a magnitude effect (Fig 2B). When the probabilities for the occurrence of an adverse effect were high (e.g., 90% vs. 45%), most of the participants chose the conservative treatment, regardless of the fact that the potency of the radical treatment can be far better (Fig 2B). The findings are consistent with the prediction of ‘loss aversion’ from prospect theory, which states that a decision-maker would overweight their loss (i.e., the occurrence of an adverse effect) [29].

### The Time-course Effect on Analgesic Decisions

Our results showed that the time-course of a treatment played a key role in the participants’ preferences. Evidence from financial decision-making has revealed that a decision-maker tends to discount the value of a choice that is delivered later, demonstrating a positive discounting rate [9,10]. Consistently, in Scenario 13 and 16 of our study, less than half of the participants favored the radical treatment, which was slow to reach its maximal effect (but with a stronger effect over the long run) (Fig 2C). Importantly, the discounting effect was moderated by the duration of delay: when the delay was rather short (e.g., for just 1 day), patients would reverse their preferences from choosing the conservative treatment to the radical one, as seen in Scenario 13/14 and 17/18 (Fig 2C). Compared to a treatment with a quick and constant effect, in these scenarios, more than half of the participants were in favor of a treatment that was slow to reach its maximal effect. In contrast to the findings of financial decision-making, our results may be attributed to the different context of the decision-making. Previous studies have demonstrated that the discount rates varied between health-related decisions and money-related decisions [30]. In the financial decision-making tasks, reward is usually delivered immediately. In contrast, in a health-related scenario, because the illness may last for a long period of time, patients may need to consider the overall long-term effect, instead of making a short-sighted choice.

### The Role of Prior Clinical Pain and Imagined Pain Relief

We found that the participants’ prior experience of clinical pain accounted for their preferences in the ‘Time-course Effect’ task (Scenario 16). A higher degree of prior clinical pain was associated with a lower probability to choose the radical treatment. In this scenario (Table 1), the radical treatment would reach its maximal effect after 5 days, which was very slow, compared to the conservative treatment. The participants who had suffered stronger clinical pain may be more impatient to wait, and thus preferred a conservative treatment, which would be quick to have an effect.

We found that individual differences in the referential pain relief accounted for the variation in individual patterns of preferences in the ‘Analgesic Effect’ task. In terms of the transition from conservatism to radicalism, the participants with a higher rating of referential pain relief were more likely to stick to the conservative treatment, regardless of the changes in the overall probability in the ‘Analgesic Effect’ task. The results are consistent with our previous findings [5], which showed that the stronger the relief the participants imagined from the conservative treatment, the weaker the relative benefits they would perceive from the radical treatment, causing them to disfavor the radical treatment. The findings imply that patients would form a prior belief about treatment effect in a relative sense, with prior clinical pain as the reference point). It highlights the importance of ‘relative relief’ in medical decision-making [14].

In contrast, individual differences in psychological traits (pain catastrophizing and behavior-approaching behavior) did not predict the variations in preferences. A potential explanation is that the present study is based on hypothetical decision-making scenarios and that no actual pain was delivered. Therefore, the driving force of fear and the motivation to relieve pain can be weakened.

Notably, we found that in the ‘Adverse Effect’ task and the ‘Time-course Effect’ task, the results from the scenarios of small and large ΔP were similar (Fig 2B and 2C). In both tasks, the participants needed to balance the benefit domain (i.e., treatment potency) with the negative domain (i.e., the occurrence of an adverse effect or a slow time-course). The results may be interpreted in terms of loss aversion, i.e., a preference of avoiding losses over obtaining gains [6]. When considering the negative domain, the participants may over-emphasize the negative effect related to a treatment (i.e. the loss), and became insensitive to the difference in ΔP (i.e. the gain). Our findings echoed the conclusions from financial decision-making, which showed that the loss was considered more significant than the gain [31].

### Limitations and Further Considerations

The conclusions of the present study should be treated with caution due to the following limitations of the study design. First, to simplify the decision-making model, we independently investigated each attribute of the treatments and assumed that all the other attributes to be equal. This assumption can be oversimplified because in a real clinical scenario, the effect of different attributes may be interwoven, and patients may focus on multiple attributes at a time (e.g., considering an overall effect of the time-course and the analgesic effect of a treatment). In multi-attribute decision-making, patients may adopt a variety of compensatory or non-compensatory strategies to make their choice [9]. These strategies are commonly seen in making health-related decisions, such as using MRI for knee surgery [32] or the management of miscarriage [33]. Because pain is both physically and emotionally suffering, patients may adopt non-compensatory strategies, such as focusing on the most desirable attributes (e.g., the potency of pain relief) and ignore all the other negative attributes. How patients make a multi-attribute choice for pain management would require further investigation.

Second, we assessed the participants’ relief of pain in a hypothetical way, i.e., to expect or imagine the treatment effect without actually experiencing that effect. We considered this approach to be ecologically more valid in terms of shared information about the treatment. For example, patients may imagine the adverse effects of a vaccination before they decide whether to receive it [34]. The pattern of preferences in such a description-based scenario may be different from that of an experience-based scenario [35]. Because in the present study the participants did not experience real pain or relief of pain, the preferences reported here should not be interpreted as an experience-based decision. Notably in clinical scenarios, patients may evaluate treatment effects and make decisions based on their prior experience of pain relief. Further investigation is required for understanding how patients make analgesic treatment-related decisions based on experience.

Third, individual differences in numeracy, including the ability to properly interpret probability information, would guide their decisions. In our study, we did not directly assess the participants’ numeracy ability. All the participants have completed an undergraduate degree, with many years of school education (Table 1), suggesting that they have a basic level of numeracy ability. Further investigation is needed to investigate the influence of individual differences in numeracy, especially the ability of interpreting probability, on decision-making.

### Clinical Implications

According to the Health Belief Model [36], health-related behavior can be guided by patients’ judgment of perceived susceptibility (i.e., the risk about getting an illness), perceived benefits (i.e., the effectiveness about reducing the threat of an illness) and perceived severity (i.e., the seriousness about getting an illness) [36]. In a real clinical scenario, patients may be biased to one of the issues, while ignoring the others. For example, non-steroidal anti-inflammatory drugs, which are often used against moderate to severe pain, can induce life-threatening allergic reactions [37]. Yet most patients would perceive the treatment to be very safe, by underestimating the susceptibility to get the adverse effect. In a different scenario, when patients overweight the severity of an adverse effect, their preferences can be disproportionally driven by the aversion of loss, i.e., the downside of a treatment, while ignoring the benefit of the treatment. Therefore, it is important for clinicians to clarify what actually motivates patients’ decisions, and being aware if their decisions are driven by a single factor.

Our findings showed that when considering the adverse effect, the participants were less sensitive to the relative potency between treatments. The findings echoed the concept loss aversion, which states that decision-makers may prefer avoiding loss to obtaining gain, and feel the loss more significant than the corresponding gain [6,31]. The findings imply that when making decisions, patients may over-emphasize the ‘cost’ of a treatment, even that treatment is relatively more potent. Consistent with our findings, a previous study has shown that, when the loss domain (the mortality rate) was emphasized, the decision-makers were less willing to take a more radical treatment, compared to the situation when the gain domain (the survival rate) was emphasized [38]. Also in a recent study on the hypothetical decision-making about health, loss aversion was identified, when the decision-makers were asked to choose between different drugs that increase life [39]. Altogether, the evidence suggest that when sharing medical information, clinicians need to be careful about the frame of description—whether an emphasis is put on the benefits or the costs would significantly influence patients’ preferences.

Furthermore, our findings echoed the recent discussion on the non-adherence behavior, such as discontinuing medication or skipping doses, in patients with chronic pain [40]. The Health Belief Model predicts that patients’ would weigh the perceived treatment benefits against its costs (e.g., adverse effects). For example, patients with psychotic symptoms may discontinue antipsychotic therapy due to adverse effect [41]. Consistently, we found subjective weighing of probability may influence their preference in pain medication. Excessively overweighing the probability to get an adverse effect (i.e., increased perceived susceptibility) could be followed by non-adherence behavior, i.e., reduced self-care and collaboration with clinicians in taking analgesic. Our findings imply that communication of the probability of an adverse effect would be critical to the non-adherence behavior of the management of chronic pain.

Finally, our findings regarding the time-course effect showed that patients may pursue a greater degree of overall effect over the long run, instead of making a myopic decision. Still, individual differences in the temporal discounting of treatment effects exist. For example, some participants were satisfied with a slow-start treatment, while the others preferred a quick-start one. This observation may apply to a chronic pain condition, in which patients usually receive a treatment that lasts for weeks or months. It is important for clinicians to differentiate patients with different preferences about the time-course of treatment.

### Conclusion

Using the analgesic decision-making tasks, we have shown that both the adverse effects and the time course of treatments guide the analgesic treatment preferences. Individual differences in prior experience of clinical pain and the degree of imagined pain relief are associated with the treatment preferences. Based on the findings, we suggest that patients may over-emphasize the negative attributes of a treatment (e.g., the occurrence of adverse effect or a slow time-course of treatment effect), which would further lead to non-adherence behavior. In terms of shared medical decision-making, both the benefits and costs of medical information should be clarified to patients.

## Supporting Information

S1 Text Detailed information about determination of sample size and the effect of the order of tasks (DOCX)
