# Supplementary material for: Preferences for Analgesic Treatments Are Influenced by Probability of the Occurrence of Adverse Effects and the Time to Reach Maximal Therapeutic Effects
Source: PLoS One. 2015 Jun 12;10(6):e0130214. doi: 10.1371/journal.pone.0130214 (PMC4466564; doi:10.1371/journal.pone.0130214)
Supplement: S1 Text — (DOCX) [file pone.0130214.s001.docx]

**Preferences for analgesic treatments are influenced by probability of the occurrence of adverse effects and the time to reach maximal therapeutic effects**

**Supporting Information**

**Determination of sample size**

The determination o the sample size was based on the statistical power analysis. The major goal of the current study is to compare the frequency for the participants to choose a radical treatment, between different scenarios. Therefore, we estimated the sample size for a test of two-tailed comparison of proportions of dependent groups, with type I error (α) controlled at 0.05 and the desired statistical power (1-β) at 0.9. The statistical power analysis was performed using G*Power 3 [[1](#_ENREF_1)]. The computation required an estimation of effect size (odds ratio, OR) and the probability π_D_ of discordant pairs, and here both OR and π_D_ were estimated based on our previous data published in [[2](#_ENREF_2)]. We calculated OR and π_D_ based on the data from Setting III (potency ΔP9→6 vs. ΔP9→0), a comparison between overall probability 90% vs. 2% [[2](#_ENREF_2)]. The setting is identical to the comparison between Scenario 4 and Scenario 6, in our current study (see Table 2 in the main text for the design of difference scenarios). Based on the conditions stated above, at least 39 participants were required.

**The effect of the order of tasks**

Across the participants, the order of the ‘Adverse Effect’ task and the ‘Time-course Effect’ task were counterbalanced. Twenty-two participants took the experiment in the order ‘Analgesic – Adverse Effect (AE) – Time-course (TC)’ (i.e. AE-TC), and 23 participants took the experiment in the order ‘Analgesic –Time-course – Adverse Effect’ (i.e. TC-AE). It should be noted that we did not adopt a full counterbalance scheme, only balancing the order of the AE and the TC tasks, which were related to our hypotheses. The first Analgesic Effect task was performed for validating our findings from our previous study [[2](#_ENREF_2)], and also for a 'warm-up' pre-test, for the participants to get familiar with the task design.

To assess the order effect, we compared the proportion that the participants to choose a radical, between the AE-TC and TC-AE groups. For each scenario, the comparison of proportions did not reveal significant difference in the proportion of radical choice, between the two order groups. We found two scenarios which results were more biased by the order effect. In Scenario 7 (Adverse Effect task), 9% and 23% of the participants chose the radical treatment, respectively, in the TC-AE and the AE-TC group. In Scenario 16 (Time-course Effect task), 57% and 32% of the participants chose the radical treatment, respectively, in the TC-AE and the A group. In both scenarios, the difference in proportions did not reach the level of significance (alpha=0.05).

**References**

1. Faul F, Erdfelder E, Lang AG, Buchner A (2007) G*Power 3: a flexible statistical power analysis program for the social, behavioral, and biomedical sciences. Behav Res Methods 39: 175-191.

2. Lin C (2013) Making the decision to stop pain: Probability and magnitude effects of expected pain relief on the choice of analgesics. Eur J Pain 17: 587-598.
